# Design, synthesis and biological evaluation of novel *N*-phosphorylated and *O*-phosphorylated tacrine derivatives as potential drugs against Alzheimer’s disease

**DOI:** 10.1080/14756366.2022.2045591

**Published:** 2022-03-31

**Authors:** Maja Przybyłowska, Krystyna Dzierzbicka, Szymon Kowalski, Sebastian Demkowicz, Mateusz Daśko, Iwona Inkielewicz-Stepniak

**Affiliations:** a Department of Organic Chemistry, Gdansk University of Technology, Gdansk, Poland; b Department of Pharmaceutical Pathophysiology, Faculty of Pharmacy, Medical University of Gdansk, Gdansk, Poland; c Department of Inorganic Chemistry, Gdansk University of Technology, Gdansk, Poland

**Keywords:** Alzheimer’s disease, tacrine, phosphorus tacrine analogs, molecular docking, hepatotoxicity, neurotoxicity, cholinesterase inhibitory activity

## Abstract

In this work, we designed, synthesised and biologically investigated a novel series of 14 *N*- and *O*-phosphorylated tacrine derivatives as potential anti-Alzheimer’s disease agents. In the reaction of 9-chlorotacrine and corresponding diamines/aminoalkylalcohol we obtained diamino and aminoalkylhydroxy tacrine derivatives. Next, the compounds were acid to give final products **6**–**13** and **16**–**21** that were characterised by ^1^H, ^13 ^C, ^31 ^P NMR and MS. The results of the docking studies revealed that the designed phosphorus hybrids, in theory can bind to AChE and BChE. All compounds exhibited significantly lower AutoDock Vina scores compared to tacrine. The inhibitory potency evaluation was performed using the Ellman’s method. The most inhibitory activity against AChE exhibited compound **8** with an IC_50_ value of 6.11 nM and against BChE **13** with an IC_50_ value of 1.97 nM and they were 6- and 12-fold potent than tacrine. Compound **19** showed the lack of hepatocytotoxicity in MTT assay.

## Introduction

Alzheimer’s disease is an age – related most common cause of dementia among elderly people. That is already the third leading cause of death after cancer and heart diseases^1^^,^^2^. According to an update to the estimates in the World Alzheimer Report 2015 (2020), there are over 50 million people worldwide living with dementia in 2020. This number will almost double every 20 years, reaching 152 million in 2050^3^. The life activity of the patients depends on the stage of disease. Mostly, at the beginning they are characterised only by mild loss of the memory, whereas in the advanced stage, they are totally dependent on someone, not able to function by their own^4^. It should be mentioned that many other symptoms occur simultaneously with AD, like depression, psychosis or cognitive impairment^5–7^. The cause of this disease is still unknown, but the main therapeutic strategies are based on cholinergic hypothesis, so available drugs are cholinesterase inhibitors^8^^,^^9^. Regarding the hydrolysis of acetylcholine by acetylcholinesterase (AChE) and butyrylcholinesterase (BChE) to choline and acetate, the concentration of this neurotransmitter in the central nervous system is insufficient to enable the proper work of brain^10–14^. Another characteristics of AD are the presence of thick extracellular β-amyloid plaques (Aβ) and intra-neuronal neurofibrillary tangles (NFTs), that lead to the death of neurons and as a result to the behavioural symptoms mentioned above^15–18^.

Despite of many efforts made by researchers from all over the world, there is still no effective drug that could stop the progression of AD.

The first drug approved by the United States Food and Drug Administration for the treatment of mild and moderate form of AD was tacrine (9-amino-1,2,3,4-tetrahydroacridine)^19^^,^^20^. It is potent and reversible inhibitor of AChE and BChE^21–23^. Despite its inhibitory potency it was withdrawn from use due to its side effects and toxicity like hepatotoxicity and gastrointestinal discomfort (nausea, vomiting, anorexia and diarrhea)^24–26^. Moreover, the bioavailability of the oral formulation was in the range of 9.9–36.4% what is a low result however, the plasma half-life remained stable by route of drug delivery. It confirms that tacrine elimination occurs by a 1st-order process^27–29^. There are evidences that showed that tacrine has additional properties such as it has influence on a reduction of β-amyloid peptide-induced apoptosis in cortical neurons^30^.

Connecting tacrine with a phosphorus moiety may affect the better penetration of cell membranes by a potential drug, stabilise the complex enzyme – inhibitor regarding taking the part of oxygen atom in phosphorus group in formation of hydrogen bonds and can enrich a compound with an additional biological properties^31^.

## Materials and methods

### General methods and materials

The procedures described below were used for the synthesis of compounds **1–14**. The rest of substrates as well as all solvents were purchased and used without further purification. Thin-layer chromatography (TLC) and preparative thin-layer chromatography (PTLC) were performed on silica gel 60 F254 plates purchased from Sigma Aldrich. Column chromatography was conducted using silica gel 60 (70**–**230 mesh) produced by Sigma Aldrich. Melting points were measured using a Stuart Scientific SMP30 apparatus. ^1^H NMR, ^13 ^C NMR and ^31 ^P NMR data were obtained on a Varian Gemini spectrometer (400 MHz for ^1^H, 125 MHz for ^13 ^C and 202 MHz for ^31 ^P NMR). Chemical shifts (δ) are reported in ppm relative to the residual peak of chloroform as a solvent (^1^H 7.26 ppm and ^13 ^C 77.16 ppm). A total proton decoupling was applied for ^13 ^C NMR spectra. For ^1^H NMR description, the following symbols are used: s (singlet), bs (broad singlet), d (doublet), t (triplet), q (quartet), quint (quintet), sext (sextet), sept (septet), m (multiplet); and the coupling constant values (*J*) are determined in Hertz. MS spectra were measured on matrix-assisted laser desorption/ionization-time on flight mass spectrometry (MALDI-TOF MS, Biflex III Bruker).

#### Synthesis of phosphorochloridic acid dibutyl ester 1

The preparation of the title compound was described by us in our previous work^32^.

#### Synthesis of 9-chloro-1,2,3,4-tetrahydroacridine 2

The procedure for the preparation of 9-chloro-1,2,3,4-tetrahydroacridine was performed according to our previous procedure^32^.

#### General procedure for the synthesis of diaminoalkyl tacrine derivatives 3-5

To a solution of 9-chlorotacrine (2 g, 9.187 mmol) in phenol (3.94 g, 41.866 mmol), the appropriate diamine (18.194 mmol) and NaI (0.186 g, 1.241 mmol) were added. The reaction was heated for 2 h at 180 °C. Afterwards, the product was dissolved in ethyl acetate and extracted with 10% aqueous KOH solution, water and twice with saturated NaCl solution. The organic layer was dried with anhydrous MgSO_4_ and concentrated in vacuum. The obtained residues were purified by liquid column chromatography in the system chloroform: methanol: amonia (15:1:0.2 v/v/v), giving the desired products (**3**–**5**).

#### N^1^*-(1,2,3,4-tetrahydroacridin-9-yl)hexane-1,6-diamine 3*

Yield 69.2%, oil; ^1^H NMR (400 MHz, CDCl_3_), δ [ppm]: 8.02–7.93 (2H, m, Ar-H), 7.61–7.54 (1H, m, Ar-H), 7.40–7.34 (1H, m, Ar-H), 5.63 (1H, s, NH), 3.65–3.56 (2H, m, CH_2_), 3.14-3.05 (2H, m, CH_2_), 2.77–2.66 (4H, m, CH_2_), 1.75–1.64 (2H, m, CH_2_), 1.55–1.35 (10H, m, CH_2_).

#### N^1^*-(1,2,3,4-tetrahydroacridin-9-yl)octane-1,8-diamine 4*

Yield 65.8%, oil; ^1^H NMR (400 MHz, CDCl_3_), δ [ppm]: 7.98 (1H, d, *J* = 8.4 Hz, Ar-H), 7.93 (1H, d, *J* = 8.3 Hz, Ar-H), 7.60–7.53 (1H, m, Ar-H), 7.39–7.33 (1H, m, Ar-H), 5.65 (1H, s, NH), 3.66–3.56 (2H, m, CH_2_), 3.12–3.04 (2H, m, CH_2_), 2.77–2.66 (4H, m, CH_2_), 1.72–1.62 (2H, m, CH_2_), 1.54–1.44 (6H, m, CH_2_), 1.37–1.24 (8H, m, CH_2_).

#### N^1^*-(1,2,3,4-tetrahydroacridin-9-yl)dodecane-1,12-diamine 5*

Yield 72.1%, oil; ^1^H NMR (400 MHz, CDCl_3_), δ [ppm]: 7.02–7.90 (2H, m, Ar-H), 7.61–7.51 (1H, m, Ar-H), 7.42–7.32 (1H, m, Ar-H), 3.56–3.44 (2H, m, CH_2_), 3.14–3.02 (2H, m, CH_2_), 2.77–2.65 (4H, m, CH_2_), 1.72–1.62 (2H, m, CH_2_), 1.54–1.44 (6H, m, CH_2_), 1.83–1.57 (8H, m, CH_2_), 1.54–1.17 (8H, m, CH_2_).

#### General method for the synthesis of phosphorus tacrine derivatives 6–13

To a solution of an appropriate dialminoalkyl tacrine derivative (0.6 mmol) (**3**–**5**) in dry pyridine (or ACN for phenyl derivatives) (1.8 mL), corresponding (diethyl/butyl/phenyl) chlorophosphate (1.2 mmol) was added dropwise. The reaction was stirred under atmosphere of nitrogen at room temperature for 24 h. Next, the solvent was removed under vacuum and the obtained residue was purified using PTLC plates in the chloroform: methanol mixture as an eluent (15:1 v/v) obtaining phosphorus tacrine analogs **6**–**13**.

#### Diethyl(6-((1,2,3,4-tetrahydroacridin-9-yl)amino)hexyl)phosphoramidate 6

Yield 51.5%, oil; ^1^H NMR (400 MHz, CDCl_3_), δ [ppm]: 8.12–8.03 (2H, m, Ar-H), 7.60–7.52 (1H, m, Ar-H), 7.39–7.31 (1H, m, Ar-H), 4.07–3.93 (4H, m, CH_2_) 3.64 (2H, t, *J* = 7.2 Hz, CH_2_), 3.44–3.37 (2H, m, CH_2_), 3.15–3.06 (2H, m, CH_2_) 2.76 (1H, br s, NH), 2.70–2.61 (2H, m, CH_2_), 1.92–1.80 (4H, m, CH_2_), 1.52–1.30 (8H, m, CH_2_), 1.05 (6H, t, *J* = 7.3 Hz, CH_3_); ^13 ^C NMR (125 MHz, CDCl_3_), δ [ppm]: 155.2, 152.9, 143.7, 129.9, 125.1, 124.2, 123.5, 118.3, 113.7, 62.2, 62.2, 48.7, 41.1, 31.6, 31.5, 31.3, 26.4, 26.2, 24.5, 22.5, 21.8, 16.2, 16.2 (d, *J_C–P_* = 7.0 Hz); ^31 ^P NMR (202 MHz, CDCl_3_), δ [ppm]: 9.17; MS found: *m/z* 434.2570 [M + H]^+^; calcd dla C_23_H_36_N_3_O_3_P: 433.25.

#### Diphenyl(6-((1,2,3,4-tetrahydroacridin-9-yl)amino)hexyl)phosphoramidate 7

Yield 39.8%, oil; ^1^H NMR (400 MHz, CDCl_3_), δ [ppm]: 8.07 (1H, t, *J* = 9.2 Hz, Ar-H), 7.53 (1H, t, *J* = 7.6 Hz, Ar-H), 7.39–7.16 (10H, m, Ar-H), 7.10 (1H, t, *J* = 7.2 Hz, Ar-H), 6.98 (1H, t, *J* = 6.8 Hz, Ar-H), 3.69 (1H, br s, NH), 3.64–3.58 (2H, m, CH_2_), 3.55–3.45 (2H, m, CH_2_), 3.07–3.01 (2H, m, CH_2_), 2.65–2.59 (2H, m, CH_2_), 1.85–1.77 (2H, m, CH_2_), 1.71–1.60 (2H, m, CH_2_), 1.51–1.26 (8H, m, CH_2_), 0.87 (1H, br s, NH); ^13 ^C NMR (125 MHz, CDCl_3_), δ [ppm]: 153.2, 153.1, 150.8, 150.8, 129.6, 129.1, 124.8, 124.2, 123, 120.3, 120,3, 120.2, 120.2, 48.4, 41.5, 31.2, 31.1, 31.0, 26.2, 25.9, 24.4, 22.5; ^31 ^P NMR (202 MHz, CDCl_3_), δ [ppm]: −10.97; MS found: *m/z* 530.2578 [M + H] ^+^; calcd for C_31_H_36_N_3_O_3_P: 529.25.

#### Diethyl(8-((1,2,3,4-tetrahydroacridin-9-yl)amino)octyl)phosphoramidate 8

Yield 64.7%, oil; ^1^H NMR (400 MHz, CDCl_3_), δ [ppm]: 8.11 (1H, d, *J* = 8.5 Hz, Ar-H), 8.06 (1H, d, *J* = 8.4 Hz, Ar-H), 7.56 (1H, t, *J* = 7.7 Hz, Ar-H), 7.35 (1H, t, *J* = 7.7 Hz, Ar-H), 4.07–3.96 (4H, m, CH_2_), 3.69–3.54 (4H, m, CH_2_), 3.16–3.08 (2H, m, CH_2_), 2.71–2.59 (4H, m, CH_2_), 1.94 (1H, s, NH), 1.91–1.81 (4H, m, CH_2_), 1.74–1.66 (2H, m, CH_2_) 1.50–1.32 (8H, m, CH_2_), 1.25 (6H, t, *J* = 7.2 Hz, CH_3_); ^13 ^C NMR (125 MHz, CDCl_3_), δ [ppm]: 155.1, 152.9, 143.5, 130.0, 125, 124.2, 123.57, 118.2, 113.5, 62.2, 48.9, 41.3, 31.6, 31.3, 29.1, 29.0, 26.7, 26.4, 24.4, 22.5, 21.8, 16.2 (d, *J_C–P_* = 7.1 Hz); ^31 ^P NMR (202 MHz, CDCl_3_), δ [ppm]: 9.15; MS found: *m/z* 462.2879 [M + H]^+^; calcd for C_25_H_40_N_3_O_3_P: 461.28.

#### Dibutyl(8-((1,2,3,4-tetrahydroacridin-9-yl)amino)octyl)phosphoramidate 9

Yield 62.8%, oil; ^1^H NMR (400 MHz, CDCl_3_), δ [ppm]: 8.17–8.08 (2H, m, Ar-H), 7.63–7.57 (1H, m, Ar-H), 7.44–7.38 (1H, m, Ar-H), 3.75–3.59 (4H, m, CH_2_), 3.45–3.36 (2H, m, CH_2_), 3.16 (2H, t, *J* = 5.8 Hz, CH_2_), 2.83–2.77 (2H, m, CH_2_), 2.70–2.65 (2H, m, CH_2_), 1.97–1.84 (4H, m, CH_2_), 1.79–1.53 (10H, m, CH_2_), 1.40–1.19 (10H, m, CH_2_), 0.98–0.93 (6H, m, CH_3_); ^31 ^P NMR (202 MHz, CDCl_3_), δ [ppm]: 8.68; MS found: *m/z* 518.2 [M + H]^+^; calcd for C_29_H_48_N_3_O_3_P: 517.34.

#### Diphenyl(8-((1,2,3,4-tetrahydroacridin-9-yl)amino)octyl)phosphoramidate 10

Yield 62.8%, oil; ^1^H NMR (400 MHz, CDCl_3_), δ [ppm]: 8.05 (1H, d, *J* = 8.8 Hz, Ar-H), 7.60–7.52 (1H, m, Ar-H), 7.40–7.18 (10H, m, Ar-H), 7.18–7.10 (1H, m, Ar-H), 7.03–6.98 (1H, m, Ar-H), 3.67–3.55 (2H, m, CH*2*), 3.31 (1H, br s, NH), 3.12–2.97 (2H, m, CH_2_), 2.85–2.80 (2H, m, CH_2_), 2.71–2.61 (2H, m, CH_2_), 1.90–1.79 (2H, m, CH_2_), 1.74–1.63 (2H, m, CH_2_), 1.50–1.30 (4H, m, CH_2_), 1.22–1.18 (8H, m, CH_2_), 0.89 (1H, br s, NH); ^13 ^C NMR (125 MHz, CDCl_3_), δ [ppm]: 153.0, 150.9, 150.8, 129.6, 129.1, 124.9, 124.1, 123.5, 123, 120.3, 120.3, 120.2, 120.2, 52.8, 48.9, 41.7, 31.4, 31.3, 29.1, 28.9, 26.6, 26.3, 24.4, 22.6, 21.9; ^31 ^P NMR (202 MHz, CDCl_3_), δ [ppm]: −10.69; MS found: *m/z* 558.2883 [M + H]^+^; calcd for C_33_H_40_N_3_O_3_P: 557.28.

#### Diethyl(12-((1,2,3,4-tetrahydroacridin-9-yl)amino)dodecyl)phosphoramidate 11

Yield 44.4%, oil; ^1^H NMR (400 MHz, CDCl_3_), δ [ppm]: 8.39 (1H, d, *J* = 8.4 Hz, Ar-H), 8.14 (1H, d, *J* = 8.6 Hz, Ar-H), 7.68 (1H, t, *J* = 7.7 Hz, Ar-H), 7.44 (1H, t, *J* = 7.7 Hz, Ar-H), 4.12–4.04 (6H, m, CH_2_), 3.80 (2H, t, *J* = 7.2 Hz, CH_2_), 3.27 (2H, t, *J* = 6.1 Hz, CH_2_), 2.94–2.85 (2H, m, CH_2_), 2.65 (2H, t, *J* = 6.0 Hz, CH_2_), 2.00–1.87 (4H, CH_2_), 1.84–1.74 (2H, m, CH_2_), 1.52–1.43 (16H, m, CH_2_), 1.20 (6H, t, *J* = 7.3 Hz, CH_3_), 0.86 (1H, br s, NH); ^31 ^P NMR (202 MHz, CDCl_3_), δ [ppm]: 9.15; MS found: *m/z* 518.4 [M + H]^+^; calcd for C_29_H_48_N_3_O_3_P: 517.34.

#### Dibutyl(12-((1,2,3,4-tetrahydroacridin-9-yl)amino)dodecyl)phosphoramidate 12

Yield 49.3%, oil; ^1^H NMR (400 MHz, CDCl_3_), δ [ppm]: 8.20 (1H, d, *J* = 8.5 Hz, Ar-H), 8.11 (1H, d, *J* = 8.7 Hz, Ar-H), 7.66–7.60 (1H, m, Ar-H), 7.45–7.37 (1H, m, Ar-H), 3.91–3.84 (4H, m, CH_2_), 3.65–3.58 (2H, m, CH_2_), 3.18 (2H, t, *J* = 5.7 Hz, CH_2_), 2.86–2.79 (2H, m, CH_2_), 2.71–2.64 (2H, m, CH_2_), 1.98–1.85 (4H, m, CH_2_), 1.83–1.54 (8H, m, CH_2_), 1.46–1.37 (4H, m, CH_2_), 1.36–1.14 (16H, m, CH_2_), 0.94 (6H, t, *J* = 7.4 Hz, CH_3_); ^31 ^P NMR (202 MHz, CDCl_3_), δ [ppm]: 1.21; MS found: *m/z* 574.3 [M + H]^+^; calcd for C_33_H_56_N_3_O_3_P: 573.41.

#### Diphenyl(12-((1,2,3,4-tetrahydroacridin-9-yl)amino)dodecyl)phosphoramidate 13

Yield 42.6%, oil; ^1^H NMR (400 MHz, CDCl_3_), δ [ppm]: 8.20 (1H, t, *J* = 6.5 Hz, Ar-H), 7.54 (1H, t, *J* = 7.7 Hz, Ar-H), 7.41–7.10 (11H, m, Ar-H), 6.99 (1H, t, *J* = 7.3 Hz, Ar-H), 3.84 (2H, t, *J* = 7.2 Hz, CH_2_), 3.34 (1H, br s, NH), 3.12–2.98 (4H, m, CH_2_), 2.61 (2H, t, *J* = 6.0 Hz, CH_2_), 1.95 (1H, s, NH), 1.88–1.69 (6H, m, CH_2_), 1.55–1.13 (18H, m, CH_2_); ^13 ^C NMR (125 MHz, CDCl_3_), δ [ppm]: 158.3, 150.9, 150.8, 129.6, 129.1, 124.9, 124.7, 124.4, 122.9, 120.3, 120.4, 120.2, 120.2, 48.3, 45.8, 41.8, 31.4, 31.4, 31.1, 29.4, 29.4, 29.1, 26.9, 26.7, 26.4, 24.1, 23.3, 22.0, 20.8; ^31 ^P NMR (202 MHz, CDCl_3_), δ [ppm]: −0.46; MS found: *m/z* 614.4 [M + H]^+^; calcd for C_37_H_48_N_3_O_3_P: 613.34.

#### General method for the synthesis of aminoalkylhydroxy tacrine derivatives 14, 15

A solution of 9-chlorotacrine (2 g, 9.19 mmol) in phenol (3.94 g, 41.87 mmol), the appropriate aminoalcohol (18.19 mmol) and NaI (0.19 g, 1.24 mmol) was heated at 180 °C for 2 h. After this time, the product was dissolved in ethyl acetate and washed with 10% aqueous KOH solution, water and twice with saturated NaCl solution. Then the organic layer was dried with anhydrous MgSO_4_ and the solvent was evaporated under vacuum. The crude product was purified with column chromatography using chloroform: methanol: amonia (20:1:0.2 v/v/v) as eluent to afford the desired aminoalkylhydroxy tacrine derivatives (**14**,**15**).

#### 3-((1,2,3,4-Tetrahydroacridin-9-yl)amino)propan-1-ol 14

Yield 59.2%, oil; ^1^H NMR (400 MHz, CDCl_3_), δ [ppm]: 8.00 (1H, d, *J* = 8.5 Hz, Ar-H), 7.92 (1H, d, *J* = 8.4 Hz, Ar-H), 7.59–7.52 (1H, m, Ar-H), 7.40–7.32 (1H, m, Ar-H), 4.52 (1H, br s, OH), 3.91 (2H, t, *J* = 5.7 Hz, CH_2_), 3.71–3.62 (2H, m, CH_2_), 3.11–3.03 (2H, m, CH_2_), 2.75 (2H, t, *J* = 5.4 Hz, CH_2_), 2.00–1.86 (6H, m, CH_2_).

#### 2-((2-((1,2,3,4-Tetrahydroacridin-9-yl)amino)ethyl)amino)ethanol 15

Yield 45.7%, oil; ^1^H NMR (400 MHz, CDCl_3_), δ [ppm]: 8.02 (1H, d, *J* = 8.4 Hz, Ar-H), 7.93 (1H, d, *J* = 8.4 Hz, Ar-H), 7.61–7.53 (1H, m, Ar-H), 7.41–7.33 (1H, m, Ar-H), 4.83 (1H, s, OH), 3.78–3.72 (2H, m, CH_2_), 3.58 (2H, t, *J* = 5.5 Hz, CH_2_), 3.12–3.05 (2H, m, CH_2_), 2.96–2.90 (2H, m, CH_2_), 2.87–2.84 (2H, m, CH_2_), 2.81–2.73 (2H, m, CH_2_), 2.02–1.88 (4H, m, CH_2_).

#### General method for the synthesis of phosphorus tacrine derivatives 16–21

An appropriate aminoalcohol (0.6 mmol) was dissolved in a dry pyridine (or ACN for phenyl derivatives) (1.8 ml) following by adding an appropriate chlorophosphate (1.2 mmol). The mixture was stirred under atmosphere of nitrogen at room temperature for 24 h. The solvent was removed under vacuum and the crude product was purified on PTLC plates in the chloroform: methanol phase (15:1 v/v).

#### Diethyl(3-((1,2,3,4-tetrahydroacridin-9-yl)amino)propyl)phosphate 16

Yield 42.9%, oil; ^1^H NMR (400 MHz, CDCl_3_), δ [ppm]: 8.28 (1H, d, *J* = 8.5 Hz, Ar-H), 8.20 (1H, d, *J* = 8.5 Hz, Ar-H), 7.60 (1H, t, *J* = 7.7 Hz, Ar-H), 7.42–7.36 (1H, m, Ar-H), 6.68 (1H, br s, NH), 4.25–4.15 (2H, m, CH_2_), 4.14–4.04 (4H, m, CH_2_), 4.02–3.92 (2H, m, CH_2_), 3.18 (2H, t, *J* = 5.7 Hz, CH_2_), 2.72 (2H, t, *J* = 5.6 Hz, CH_2_), 2.23–2.14 (2H, m, CH_2_), 1.90–1.80 (4H, m, CH_2_), 1.30 (6H, t, *J* = 7.1 Hz, CH_3_); ^13 ^C NMR (125 MHz, CDCl_3_), δ [ppm]: 154.6, 153.0, 140.8, 131.2, 124.8, 113.8, 122.6, 117.0, 112.8, 64.8, 64.2, 64.1, 44.6, 31.3, 29.8, 24.5, 22.3, 21.2, 16.1 (d, *J_C–P_* = 6.6 Hz); ^31 ^P NMR (202 MHz, CDCl_3_), δ [ppm]: −0.45; MS found: *m/z* 393.1 [M + H]^+^; calcd for C_20_H_29_N_2_O_4_P: 392.19.

#### Dibutyl(3-((1,2,3,4-tetrahydroacridin-9-yl)amino)propyl)phosphate 17

Yield 45.1%, oil; ^1^H NMR (400 MHz, CDCl_3_), δ [ppm]: 8.54 (1H, d, *J* = 8.4 Hz, Ar-H), 8.45 (1H, d, *J* = 8.3 Hz, Ar-H), 7.74–7.63 (2H, m, Ar-H), 4.19–4.06 (8H, m, CH_2_), 3.79–3.73 (2H, m, CH_2_), 3.37–3.30 (2H, m, CH_2_), 3.28 (2H, t, *J* = 6.0 Hz, CH_2_), 2.44–2.35 (2H, m, CH_2_), 2.25–2.16 (2H, m, CH_2_), 1.33–1.22 (8H, m, CH_2_), 0.97–0.93 (6H, m, *J* = 7.3 Hz, CH_3_); ^31 ^P NMR (202 MHz, CDCl_3_), δ [ppm]: 0.38; MS found: *m/z* 449.2 [M + H]^+^; calcd for C_24_H_37_N_2_O_4_P: 448.25.

#### Diphenyl(3-((1,2,3,4-tetrahydroacridin-9-yl)amino)propyl)phosphate 18

Yield 47.7%, oil; ^1^H NMR (400 MHz, CDCl_3_), δ [ppm]: 8.07–7.99 (2H, m, Ar-H), 8.63–7.55 (1H, m, Ar-H), 7.45–7.37 (1H, m, Ar-H), 7.32–7.01 (10H, m, Ar-H), 3.75 (2H, t, *J* = 6.8 Hz, CH_2_), 3.68 (2H, t, *J* = 6.2 Hz, CH_2_), 3.15–3.08 (2H, m, CH_2_), 2.78–2.70 (2H, m, CH_2_), 2.22–2.13 (2H, m, CH_2_), 1.98–1.86 (4H, m, CH_2_); ^31 ^P NMR (202 MHz, CDCl_3_), δ [ppm]: −11.28; MS found: *m/z* 489.1 [M + H]^+^; calcd for C_28_H_29_N_2_O_4_P: 488.19.

#### Diethyl(2-((2-((1,2,3,4-tetrahydroacridin-9-yl)amino)ethyl)amino)ethyl)phosphate 19

Yield 39.9%, oil; ^1^H NMR (400 MHz, CDCl_3_), δ [ppm]: 8.49 (1H, d, *J* = 8.5 Hz, Ar-H), 8.33 (1H, d, *J* = 8.7 Hz, Ar-H), 7.66 (1H, t, *J* = 7.7 Hz, Ar-H), 7.47–7.40 (1H, m, Ar-H), 7.19 (1H, s, NH), 4.20–3.99 (6H, m, CH_2_), 3.68–3.58 (2H, m, CH_2_), 3.39–3.22 (4H, m, CH_2_), 2.76 (2H, t, *J* = 5.6 Hz, CH_2_), 1.95–1.81 (4H, m, CH_2_), 1.39 (2H, t, *J* = 7.3 Hz, CH_2_), 1.32–1.25 (7H, m, CH_3_+NH); ^13 ^C NMR (125 MHz, CDCl_3_), δ [ppm]: 155.7, 152.0, 139.3, 131.8, 124.9, 124.0, 121.5, 116.2, 111.5, 65.8, 64.1, 63.2, 48.6, 47.4, 45.8, 28.8, 24.5, 22.2, 20.8, 16.2 (d, *J_C–P_* = 7.0 Hz); ^31 ^P NMR (202 MHz, CDCl_3_), δ [ppm]: −0.96; MS found: *m/z* 422.1 [M + H]^+^; calcd for C_21_H_32_N_3_O_4_P: 421.21.

#### Dibutyl(2-((2-((1,2,3,4-tetrahydroacridin-9-yl)amino)ethyl)amino)ethyl)phosphate 20

Yield 41.7%, oil; ^1^H NMR (400 MHz, CDCl_3_), δ [ppm]: 8.48 (1H, d, *J* = 8.7 Hz, Ar-H), 8.21 (1H, d, *J* = 8.6 Hz, Ar-H), 7.71–7.66 (1H, m, Ar-H), 7.47–7.40 (1H, m, Ar-H), 4.17–3.98 (4H, m, CH_2_), 3.97–3.89 (2H, m, CH_2_), 3.80 (2H, t, *J* = 5.7 Hz, CH_2_), 3.28 (2H, t, *J* = 5.9, CH_2_), 3.23–3.16 (2H, m, CH_2_), 2.65 (2H, t, *J* = 6.0, CH_2_), 1.98–1.81 (6H, m, CH_2_), 1.74–1.62 (4H, m, CH_2_), 1.34–1.22 (4H, m, CH_2_), 0.95 (6H, m, CH_3_); ^31 ^P NMR (202 MHz, CDCl_3_), δ [ppm]: 0.07; MS found: *m/z* 478.2 [M + H]^+^; calcd for C_25_H_40_N_3_O_4_P: 477.28.

#### Diphenyl(2-((2-((1,2,3,4-tetrahydroacridin-9-yl)amino)ethyl)amino)ethyl)phosphate 21

Yield 46.8%, oil; ^1^H NMR (400 MHz, CDCl_3_), δ [ppm]: 8.70–8.58 (1H, m, Ar-H), 8.25–8.17 (1H, m, *J* = Ar-H), 7.74–7.66 (1H, m, Ar-H), 7.51 (1H, t, *J* = 7.7 Hz, Ar-H), 7.39–7.20 (10H, m, Ar-H), 4.56–4.45 (2H, m, CH_2_), 4.08–3.97 (2H, m, CH_2_), 3.78–3.61 (4H, m, CH_2_), 3.07–2.98 (2H, m, CH_2_), 2.49–2.38 (2H, m, CH_2_), 1.72–1.57 (4H, m, CH_2_); ^13 ^C NMR (125 MHz, CDCl_3_), δ [ppm]: 155.3, 150.3, 149.6, 129.9, 129.8, 125.6, 124.9, 123.9, 123.1, 120.1, 120.1, 119.9, 119.9, 66.8, 48.1, 47.6, 45.8, 28.4, 23.9, 21.9, 20.6; ^31 ^P NMR (202 MHz, CDCl_3_), δ [ppm]: −12.03; MS found: *m/z* 518.2 [M + H]^+^; calcd for C_29_H_32_N_3_O_4_P: 517.21.

### Biological studies

#### Cell culture

Human neuroblastoma SH-SY5Y and human liver hepatocellular HepG2 cell lines were purchased from the American Type Culture Collections (ATCC). SH-SY5Y cells were cultured in a Dulbecco’s Modified Eagle Medium (DMEM) supplemented with Ham F12 medium (ratio 1:1), whereas HepG2 cells in Eagle’s Minimum Essential Medium (EMEM). Additionally, all culture medium was supplemented with 10% foetal bovine serum (FBS), streptomycin (100 µg/mL) and penicillin (100 U/mL). The cells were seeded into flasks containing supplemented medium and maintained in a humidified atmosphere of 5% CO_2_ and 95% air at 37 °C. The cell viability assay was performed in confluence of the cells in serum free medium (without FBS) was 80–90%.

#### Cell viability assay

The potential neurocytotoxic and hepatocytotoxic effect of the new tacrine derivatives was examined by the 3-(4,5-dimethylthiazol-2-yl)-2,5-diphenyl tetrazolium bromide (MTT) test according to the commercially available protocol (Abcam Cambridge, MA). SH-SY5Y cells and HepG2 cells were seeded on 96-well plate with a density of 27–29 × 10^3^ cells/well and 12–14 × 10^3^/well respectively. After 24 h of incubation, the cells were incubated for 24 h with newly synthesised analogs (and tacrine as the reference) in the range of concentrations of 25–600 µM. All of tested compounds were dissolved in DMSO, diluted in serum-free medium. The DMSO concentration of every well in final dilutions was equal to or less than 0.1%. After incubation with compounds, MTT was added and 2 h later, the absorbance was measured at 492 nm. The result was expressed as the percentage of control cells (cultured in serum-free medium containing 0.1% of DMSO) which was set to 100%. The MTT test was performed in three independent replications. Data analysis was performed by using GraphPad Prism 5.0 Software.

#### AChE and BChE inhibitory activity

Acetylcholinesterase (eeAChE, E.C. 3.1.1.7, from the electric eel), butyrylcholinesterase (BChE, E.C. 3.1.1.8, from equine serum), 5,5′-dithiobis-(2-nitrobenzoic) acid (Ellman’s reagent, DTNB), acetylthiocholine iodide (ATCh), butylthiocholine iodide (BTCh) and tacrine hydrochloride were obtained from Sigma Aldrich company. The inhibition of mentioned enzymes was determined for tested compounds in the range of concentrations of 1–1000 nM by dissolving them in DMSO and diluting in phosphate buffer (pH 7.5, 0.1 M). ^33^ Inhibitory activity was tested at room temperature on 96-well plate using Ellman’s method^33^. Lyophilised powders of AChE and BChE were dissolved in phosphate buffer (pH 7.5, 0.1 M) with 1% BSA.

The absorbance was recorded at the wavelength of 412 nm. Each well was filled to a total volume of 330 µL. The content inculded 285 µL of phosphate buffer, 5 µL 2 units/mL of eeAChE/BChE, 10 µL 0.01 M of DTNB, 10 µl of tested compound (for the control wells 10 µL of the phosphate buffer instead of tested compound) and 20 µL (7.5 mM) ATCh/BTCh. The hydrolysis of ATCh/BTCh was initiated by adding it to the well directly before the measurement. The absorbance was recorded at 1 min intervals for 8 min in total. The percent of inhibition was calculated by nonlinear regression analysis of the response–concentration (log) curve as a percentage of the reaction rate of the tested compounds over the control. Data analysis was performed by using GraphPad Prism 5.0 Software to give the IC_50_ values. The experiment was carried out in triplicate.

#### Molecular modelling studies

All the molecular structures of the ligands were built with the program Portable HyperChem 8.0.7 Release (Hypercube, Inc., Gainesville, FL) and were energy minimised using the MM + force field and Polak – Ribiere conjugate gradient algorithm. The iteration procedure was continued until energy gradients became less than 0.05 kcal/mol/Å. The X-ray structures of the AChE and BChE enzymes used for molecular modelling studies were taken from the Protein Databank (Protein Data Bank accession codes: 2CMF and 1P0I, respectively). After standard preparation procedures (including removal of water molecules and other ligands as well as addition hydrogen atoms and Gasteiger charges to each atom) docking analysis was carried out. A docking study was carried out using Autodock Vina 1.1.2 software (The Molecular Graphic Laboratory, The Scripps Research Institute, La Jolla, CA)^34^. For the docking studies to the AChE enzyme a grid box size of 30 Å × 30 Å × 30 Å centred on Phe330 amino acid residue was used. For the docking studies to the BChE enzyme a grid box size of 30 Å × 30 Å × 30 Å centred on Phe329 amino acid residue was used. Graphic visualisations of the 3 D model were generated using VMD 1.9 (the University of Illinois at Urbana – Champaign, Urbana, IL).

## Results and discussion

### Chemistry

**Scheme 1.**
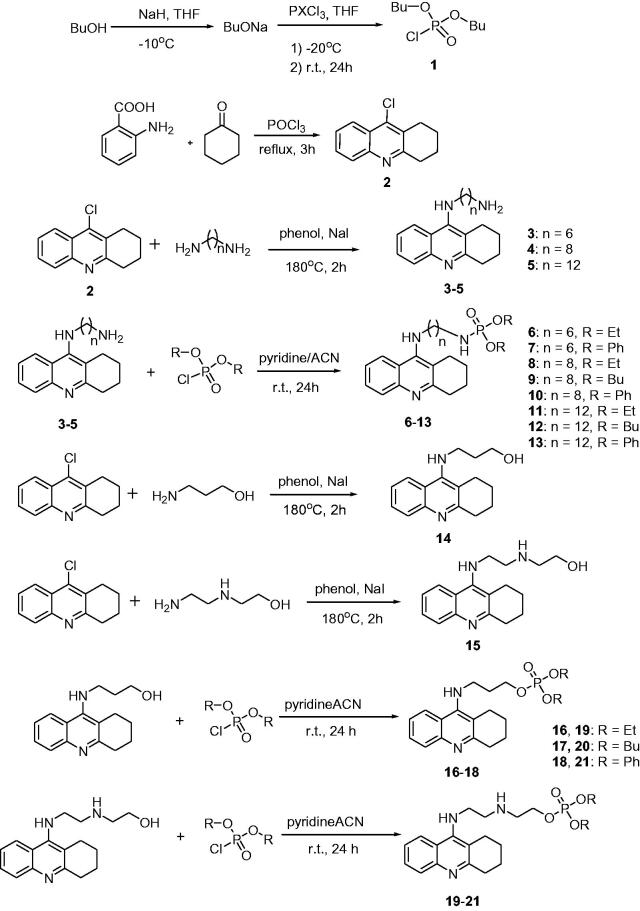
Synthesis of new phosphorus tacrine derivatives **6**–**13**, **16**–**21**.

The synthetic route leading to the final compounds **6**–**13** and **16**–**21** is presented in Scheme 1. The first step included the preparation of phosphorochloridic acid dibutyl ester from butan-1-ol and sodium hydroxide, what was immediately transformed into ester by adding POCl_3_. Both reactions were performed in THF. Next, 9-chlorotacrine was obtained from cyclohexanone, anthranilic acid and POCl_3_. Then, that was connected with appropriate diamine or aminoalcohol (in the presence of sodium iodide) in phenol to get diaminoalkyl and aminoalkylhydroxy tacrine derivatives. The last stage was performed in the reaction of synthesised tacrine analogs mentioned in the previous step and corresponding phosphorochloridic acid esters. Final products were described by MS, ^1^H NMR, ^31 ^P NMR and where that was possible by ^13 ^C NMR.

### Molecular modelling

To examine the possible interactions of newly designed phosphorus tacrine analogs with amino acid residues within the active site of AChE and BChE, potential AChE/BChE inhibitors were docked into the crystal structure of the acetylcholinesterase (Protein Data Bank accession code 2CMF) and butyrylcholinesterase (Protein Data Bank accession code 1P0I). The procedures for docking analyses as well as protein and inhibitor preparations were described in detail in the experimental section. After standard preparation procedures (including removal of water molecules and other ligands as well as the addition of hydrogen atoms and Gasteiger charges to each atom) docking analysis was carried out using Autodock Vina 1.1.2 software (The Molecular Graphic Laboratory, The Scripps Research Institute, La Jolla, CA).

Our docking experiments revealed that newly designed phosphorus tacrine analogs could, at least theoretically, possess AChE and BChE-binding ability. All compounds expressed satisfactory predicted free docking energies (in the range of −9.2 to −12.5 kcal/mol for AChE and −7.8 to −10.9 kcal/mol for BChE) and exhibited significantly lower AutoDock Vina scores compared to a reference tacrine **16** (−9.0 kcal/mol for AChE and −8.2 kcal/mol for BChE) (see Table 1). Furthermore, free binding energy analysis showed that in the case of compounds **1**–**9**, the best accommodation was found for tacrine analogs having linkers containing from 6 and 8 carbon atoms. The docking of compounds with longer chains led to an increase in predicted free binding energies, which is associated with lower stability of the enzyme–inhibitor complex. These outcomes may suggest that compounds with the linkers longer than 8 atoms seem to be too extensive.

**Table 1.** Free binding energies calculated for potential AChE/BChE inhibitors **6**–**21** and reference tacrine **16**.

No.	Structure	AChE Free binding energy [kcal/mol]	BChE Free binding energy [kcal/mol]
**6**	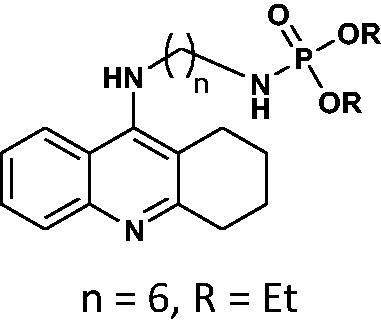	−10.1	−8.7
**7**	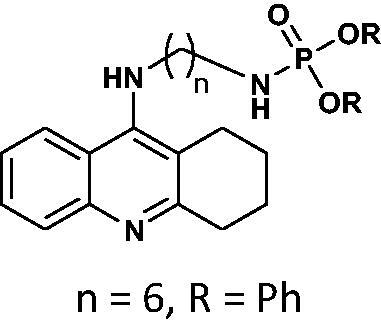	−12.5	−10.7
**8**	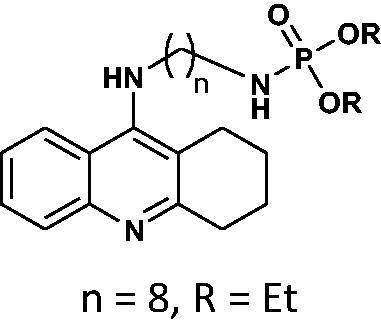	−10.4	−8.0
**9**	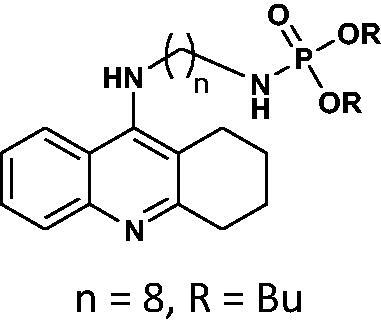	−10.2	−8.0
**10**	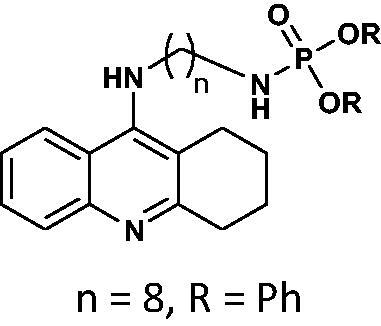	−11.9	−10.3
**11**	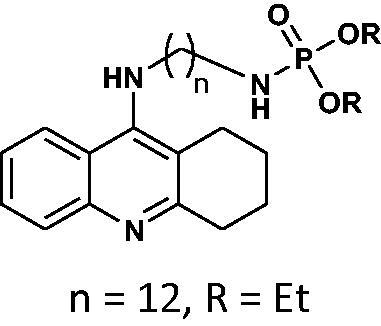	−9.8	−7.8
**12**	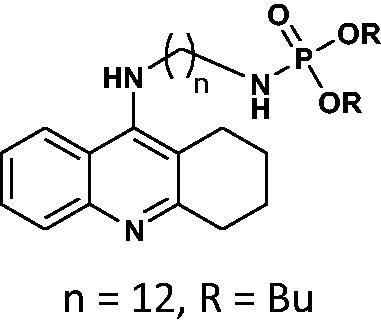	−9.2	−7.8
**13**	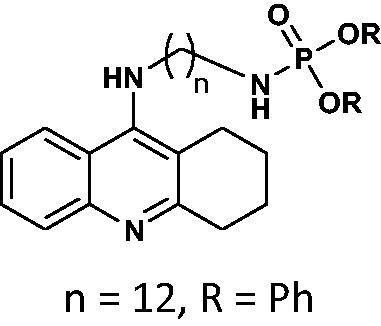	−11.1	−9.9
**16**	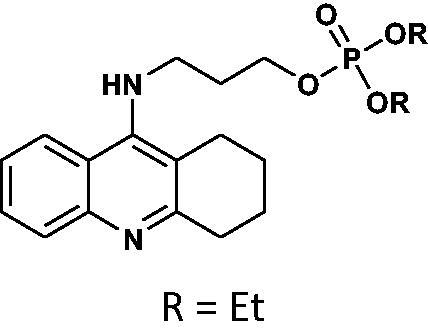	−9.5	−8.1
**17**	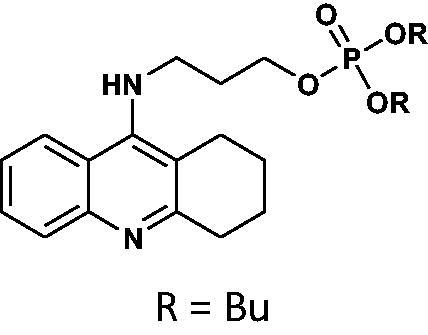	−9.5	−8.6
**18**	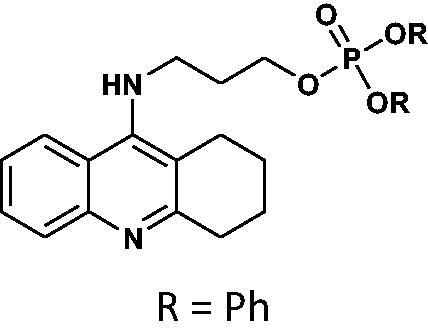	−12.1	−10.6
**19**	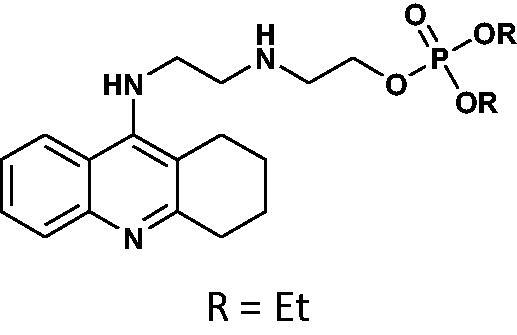	−9.6	−8.1
**20**	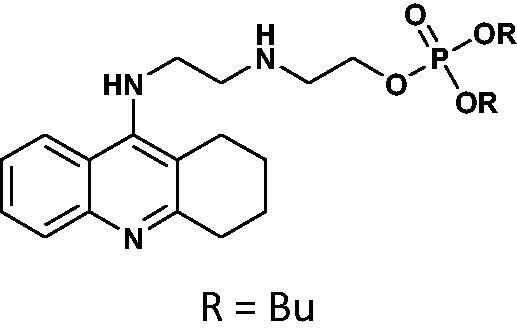	−9.8	−8.5
**21**	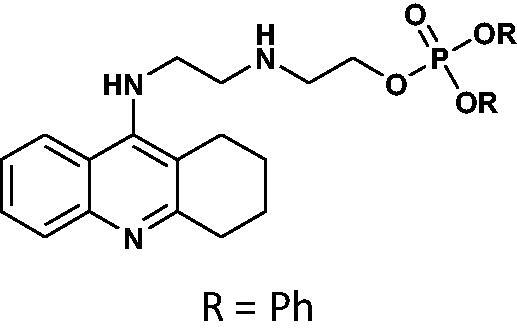	−12.1	−10.6
**tacrine**	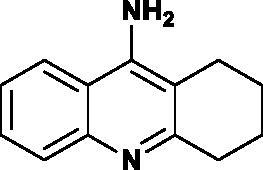	−9.0	−8.2

**Figure 1.**
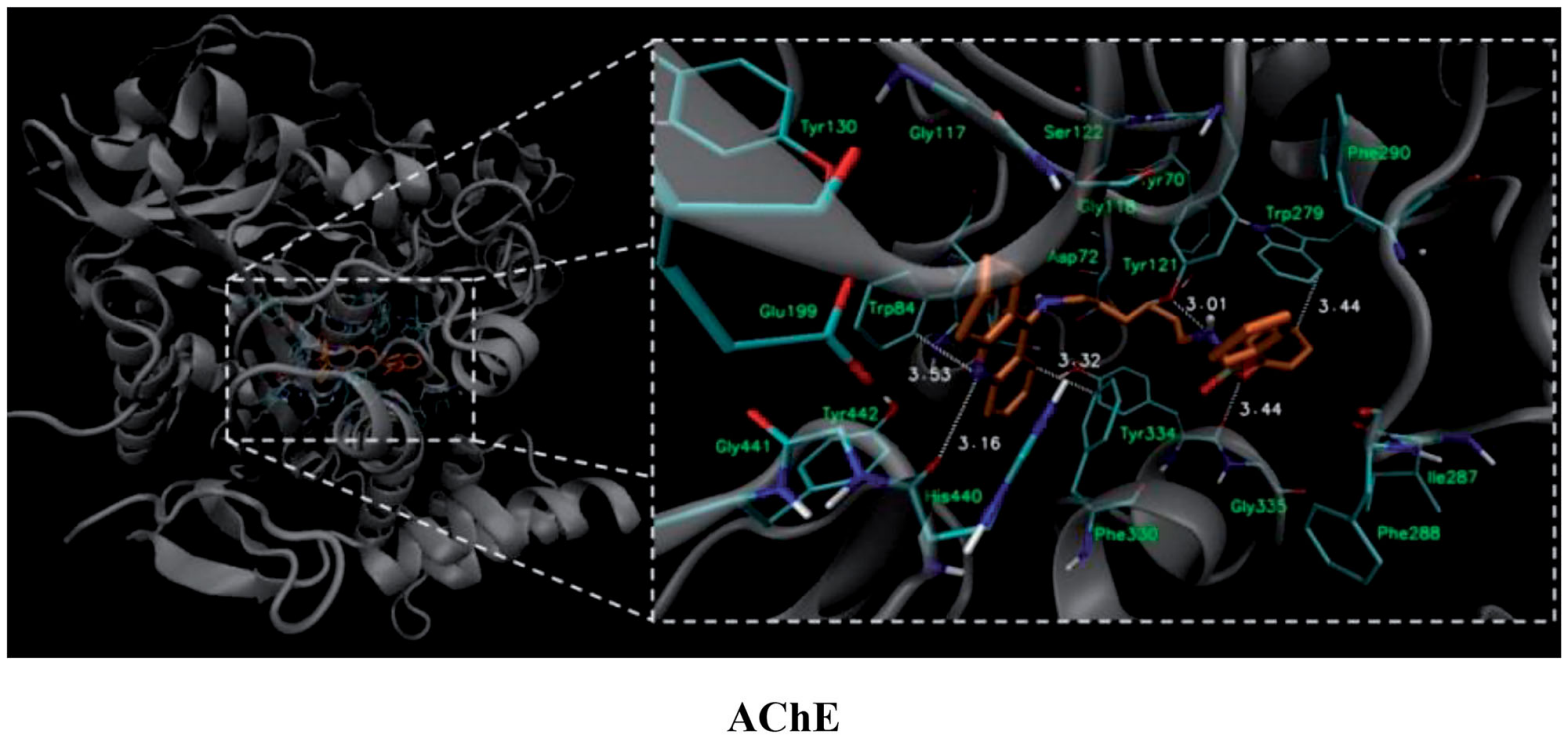
Docked binding modes for compound **7** and AChE.

**Figure 2.**
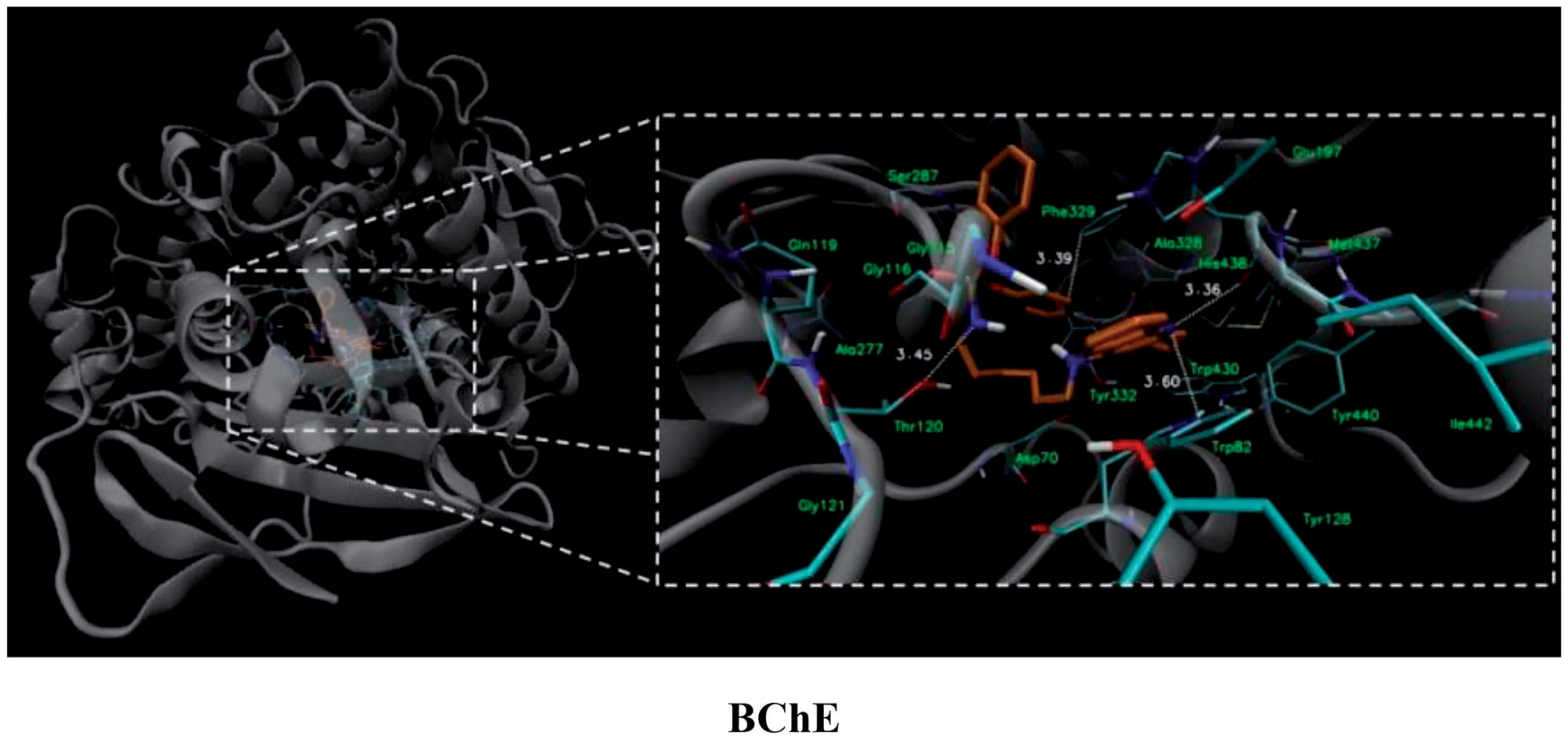
Docked binding modes for compound **7** and BChE.

For compound **7**, in general, the results of the docking studies predicted the best binding poses towards the catalytic cavity of AChE and BChE, expressed in terms of docking energies of −12.5 and −10.7 kcal/mol for AChE and BChE, respectively. A network of potential interactions was established between the phosphoramidate tacrine analog **7** and the neighbouring amino acid residues creating key enzyme regions of both enzymes such as catalytic active site (CAS), midgorge, and peripheral anionic site (PAS). For example, within the CAS region of both enzymes π-π stacking interactions between the tacrine aromatic ring and Phe330, Trp84 (AChE) and Trp82 (BChE) and the hydrogen bonding between the tacrine nitrogen atom and His440 (AChE) and His438 (BChE) were found. Moreover, the introduction of the phosphorus moiety may affect the additional stabilisation of the inhibitor-enzyme complex as a result of the ability to form the hydrogen bonds between phosphoramidate group and Tyr121 (AChE) and Thr120 (BChE) in the midgorge regions as well as the possibility to occurrence of non-covalent interactions including π–π stacking of phenyl ester function with Trp279 (in the PAS of AChE). Docked binding modes for compound **7** are presented in Figures 1 and 2.

### Biological activity

#### Cell viability assays

##### SH-SY5Y cell line

SH-SY5Y a standard cell line used for determining neurocytotoxic effect of potential drug. Moreover it is well-established *in vitro* model of neurodegenerative disorders^35–37^. Therefore, have used it in our research to establish a neurotoxicity of newly synthesised tacrine analogs. They were tested in the range of concentrations of 12.5 − 100 µM.

Almost all of the derivatives, that consist only nitrogen atoms in the linker between tacrine and phosphorus moiety were more neurocytotoxic than a tacrine (reference). Only analog **6** exhibited similar to tacrine cytotoxic effect against SH-SY5Y cells at the concentration of 100 µM, caused a decrease in the cell viability to 67.49%, compared to 68.90% for tacrine. The calculated results for the rest of analogs at the same 100 µM concentration were in the range of 30.11–54.65%. All results were shown in Table 2.

**Table 2.** Cells viability of SH-SY5Y cell line after incubation with compounds **6–13** and tacrine for 24 h (% of control).

Compounds	12.5 µM	50 µM	100 µM
**6**	74.32 ± 4.9	71.72 ± 7.2*	67.49 ± 9.0 **
**7**	47.36 ± 1.2***	41.26 ± 1.5***	30.11 ± 5.0***
**8**	76.70 ± 5.8**	51.58 ± 5.4***	52.25 ± 1.0
**9**	77.51 ± 13.5	55.69 ± 11.5**	54.65 ± 8.4**
**10**	80.09 ± 16.8	63.70 ± 5.7*	48.07 ± 8.1***
**11**	35.40 ± 4.6***	35.50 ± 4.1***	31.15 ± 6.4***
**12**	69.34 ± 3.7***	52.11 ± 3.1***	53.53 ± 5.8***
**13**	40.36 ± 7.6*	38.35 ± 7.6*	37.98 ± 3.8*
**Tacrine**	98.74 ± 4.5	97.25 ± 2.1	68.90 ± 2.9 ***

Means ± SD from triplicates from three different experiments. *p < 0.05; **p < 0.01; ***p < 0.001, as compared to the control.

Among the series of compounds with an oxygen atom in the linker two of them, **16** and **19**, proved to be much less cytotoxic than tacrine (cell viability was 83.5% and 81.1%, respectively). Additionally, **21** was about two times more toxic than tacrine at concentration of 100 µM whereas the rest analogs exhibited similar neurotoxic effect. The results were shown in Table 3.

**Table 3.** Cells viability of SH-SY5Y cell line after incubation with compounds **16**–**21** and tacrine for 24 h (% of control).

Compounds	12.5 µM	50 µM	100 µM
**16**	88.06 ± 7.8	80.04 ± 7.7	83.47 ± 8.6
**17**	94.42 ± 5.1	67.73 ± 14.7**	63.18 ± 12.2**
**18**	79.50 ± 7.4	71.25 ± 20.5	58.99 ± 20.3*
**19**	85.46 ± 12.6	82.59 ± 3.2*	81.12 ± 5.0
**20**	78.91 ± 8.6*	79.69 ± 1.1*	68.63 ± 14.5***
**21**	52.27 ± 5.0***	42.72 ± 6.4***	30.67 ± 1.5***
**Tacrine**	98.74 ± 4.5	97.25 ± 2.1	68.90 ± 2.9***

Means ± SD from triplicates from at least three different experiments. *p < 0.05; **p < 0.01; ***p < 0.001, as compared to the control.

Our results for the least neurocytotoxic analogs are different from these reported by Scipioni et al.^38^, where the most promising tacrine derivatives adecreased cell viability of SH-SY5Y cell line to more than 50% at concentration of 100 µM. Furthermore, Oukoloff et al.^39^ evaluated a neurocytotoxicity on the same cell line for three compounds. All of them showed lower than 60% of cell viability at the concentration of 100 µM.

##### HepG2 cell line

Tacrine was withdrawn from clinic use because of its hepatotoxic properties, so it is highly desirable to reduce or even exclude the toxic effect on the liver of newly synthesised analogs^40^. HepG2 is a commonly used *in vitro* model for the investigation of hepatocytotoxic effect of xenobiotics ^40^^,^^41^. In our study, we evaluated the toxicity of our new analogs against Hep2 cells. Among of compounds with nitrogen atoms all of them were more toxic against the HepG2 cells than tacrine (Table 4).

**Table 4.** Cytotoxicity of compounds **6**–**13** on HepG2 cells.

Compounds	IC_50_ [µM]
**6**	140.2
**7**	14.6
**8**	23.7
**9**	43.3
**10**	7.15
**11**	0.05
**12**	20.77
**13**	–
**Tacrine**	189.9

The IC_50_, was calculated from the following equation: log (inhibitor) versus responses curve using the GraphPad Prism 5 program.

Among the hybrids with oxygen atom in the linker, only compound **19** showed the significant reduction of hepatotoxicity against HepG2 cells (IC_50_ value was >600 µM), whereas tacrine revealed the IC_50_ value of 189.9 µM (Table 5).

**Table 5.** Cytotoxicity of compounds **16**–**21** and tacrine on HepG2 cells.

Compounds	IC_50_ [µM]
**16**	113.9
**17**	72.28
**18**	53.83
**19**	>600
**20**	134.5
**21**	–
**Tacrine**	189.9

The IC_50_, was calculated from the following equation: log (inhibitor) versus responses curve using the GraphPad Prism 5 program.

Eghtedari et al.^14^ synthesised a series of new tacrine-derivatives, where two of them exhibited a moderate hepatotoxicity Furthermore, Chioua et al.^42^ described a series of tacripyrimidynes, where almost all of them were cytotoxic against HepG2 in the same range or higher like tacrine.

##### AChE/BChE inhibitory activity

We established profile of esterases inhibition for all of 14 synthesised compounds. We described an inhibitory potency by calculated IC_50_ values (Table 6). Tacrine was used as a reference compound. Both of enzyme, which we used in our investigation, AChE and BChE, come from the electric eel and equine serum respectively. However, they are commonly used instead of human enzymes because of high sequence identity ^43^. The most inhibitory activity against AChE had compounds **6**, **8**, **9** and **11**–**13**, where derivatives **6** and **11** showed similar activity to tacrine and the rest of mentioned analogs exhibited IC_50_ values in the range of 6.11–16.34 nM. IC_50_ value calculated for a tacrine was equal to 35.12 nM and for the strongest analog **8** was 6.11 nM, what means it is almost 6 times more potent.

**Table 6.** Inhibition of eeAChE and BChE enzymes and selectivity by tacrine and its new derivatives **6**–**13** and **16**–**21**.

Compounds	IC_50_, nM (pIC_50_ ± S.E.M.) AChE	IC_50_, nM (pIC_50_ ± S.E.M.) BChE	Selectivity IC_50_(eeAChE)/ IC_50_(BChE)
**Tacrine**	35.12	23.53	1.49
	(1.382 ± 0.026)	(1.372 ± 0.041)	
**6**	32.58	6.753	4.82
	(1.513 ± 0.026)	(0.83 ± 0.087)	
**7**	125.0	41.13	3.04
	(0.786 ± 0.025)	(1.614 ± 0.043)	
**8**	6.110	12.86	0.48
	(2.097 ± 0.042)	(1.109 ± 0.055)	
**9**	8.18	41.49	0.2
	(0.913 ± 0.027)	(1.618 ± 0.038)	
**10**	44.24	188.4	0.24
	(1.646 ± 0.087)	(2.275 ± 0.042)	
**11**	33.27	65.45	0.51
	(1.522 ± 0.052)	(1.816 ± 0.89)	
**12**	16.34	61.17	0.27
	(1.213 ± 0.07)	(1.787 ± 0.078)	
**13**	9.955	1.969	5.06
	(0.998 ± 0.101)	(0.294 ± 0.03)	
**16**	245.6	17.10	14.36
	(2.390 ± 0.159)	(1.233 ± 0.066)	
**17**	110.0	29.11	3.78
	(2.041 ± 0.034)	(1.464 ± 0.077)	
**18**	58.61	37.74	1.55
	(1.768 ± 0.032)	(2.577 ± 0.049)	
**19**	676.7	111.3	6.08
	(2.830 ± 0.063)	(2.05 ± 0.04)	
**20**	81.56	186.2	0.44
	(1.911 ± 0.026)	(1.10 ± 0.05)	
**21**	501.3	41.03	12.22
	(2.70 ± 0.110)	(1.613 ± 0.056)	

Among twelve analogs evaluated by Fancellu et al.^15^ only three were more active than tacrine and the strongest one exhibited three times higher inhibitory potency against eeAChE. Moreover, Wu et al.^44^ examined biologically tacrine-triazole derivatives and the most potent one proved to be only two times stronger than tacrine, whereas our strongest analog was six times more potent in comparison to tacrine. Moreover, our newly-synthesised analogs were shown significant inhibitory activity against BChE

IC_50_ value calculated for tacrine was 23.53 nM however, our four tested compounds revealed higher inhibitory potency against BChE namely analogs **6**, **8**, **13** and **16** with IC_50_ values of 6.75, 12.86, 1.97 and 17.10 nM, respectively. The most potent inhibitor revealed 12 times higher activity than tacrine. Makhaeva et al.^43^ reported tacrine and 1,2,4-thiadiazole derivatives, where four of them more strongly inhibited BChE activity than tacrine. However, the most active compound was almost 8 times more active. Furthermore, Derabli et al.^45^ synthesised 12 novel tacrine-pyranopyrazole hybrids, but neither of them had stronger inhibitory activity against BChE than tacrine. Similar result was obtained by Digiacomo et al.^46^, where was no more active compound than tacrine.

Inhibition curves for AChE and BChE for two the most promising compounds (base on analysis of cytotoxic properties of each compound and inhibitory activity), **6** and **19**, are presented in Figures 3 and 4, respectively (Supplement).

## Conclusions

As a continuation of our previously reported research^32^, we designed, synthesised and biologically evaluated the series of new phosphorus tacrine derivatives containing diaminoalkyl and aminohydroxyalkyl linker between these two moieties. A biological evaluation included determination of potential neurotoxic and hepatotoxic effect as well as an investigation of inhibitory activity against AChE and BChE. The derivatives with the linker consisting an oxygen atom were less toxic against SH-SY5Y cells than derivatives with nitrogen atoms. Moreover, the reduction of hepatotoxicity exhibited analog **19**. The most potent AChE inhibitors were compounds **8** and **9**, which were six and four times more potent, respectively, than tacrine. The most active against BChE were derivatives **6** and **13** exhibiting three and twelve times higher activity than a tacrine.

Presented data confirmed a therapeutic potential of our newly-synthesised tacrine analogs. Nevertheless, further structural alterations of chemical structure and more in-depth biological research are needed.

## Supplementary Material

Supplemental Material
