# Supplementary material for: Design, synthesis and biological evaluation of novel N-phosphorylated and O-phosphorylated tacrine derivatives as potential drugs against Alzheimer’s disease
Source: J Enzyme Inhib Med Chem. 2022 Mar 31;37(1):1012–22. doi: 10.1080/14756366.2022.2045591 (PMC8979514; doi:10.1080/14756366.2022.2045591)

Design, synthesis and biological evaluation of novel *N*-phosphorylated and *O*-phosphorylated tacrine derivatives as potential drugs against Alzheimer's disease

Maja Przybyłowska[a], Krystyna Dzierzbicka[a*], Szymon Kowalski[b], Sebastian Demkowicz[a], Mateusz Daśko[c], Iwona Inkielewicz-Stepniak[b*],

[a] Department of Organic Chemistry, Gdansk University of Technology, G. Narutowicza 11/12, PL-80-233 Gdansk, Poland
[b] Department of Pharmaceutical Pathophysiology, Faculty of Pharmacy, Medical University of Gdansk, Debinki 7, PL-80-211 Gdansk, Poland
[c] Department of Inorganic Chemistry, Gdansk University of Technology, G. Narutowicza 11/12, PL-80-233 Gdansk, Poland

CONTACT*:* Krystyna Dzierzbicka (krydzier@pg.edu.pl.), G. Narutowicza 11/12, 80-233 Gdansk, Poland or Iwona Inkielewicz-Stepniak (iinkiel@gumed.edu.pl.), Debinki 7, PL-80211 Gdansk, Poland

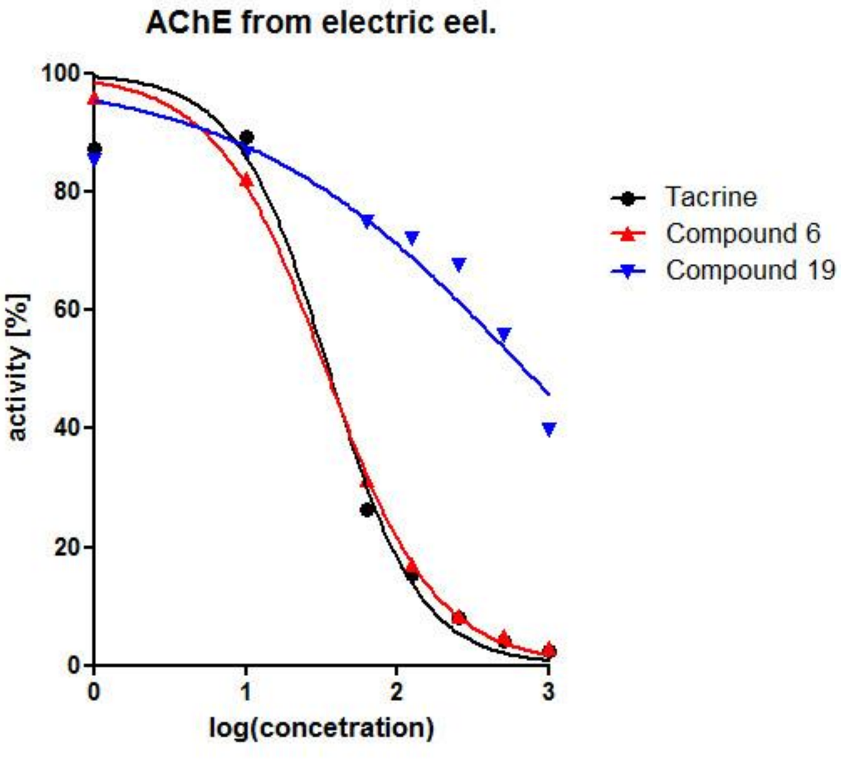

Figure 3. Inhibition curves of compounds **6** and **19** against eeAChE.

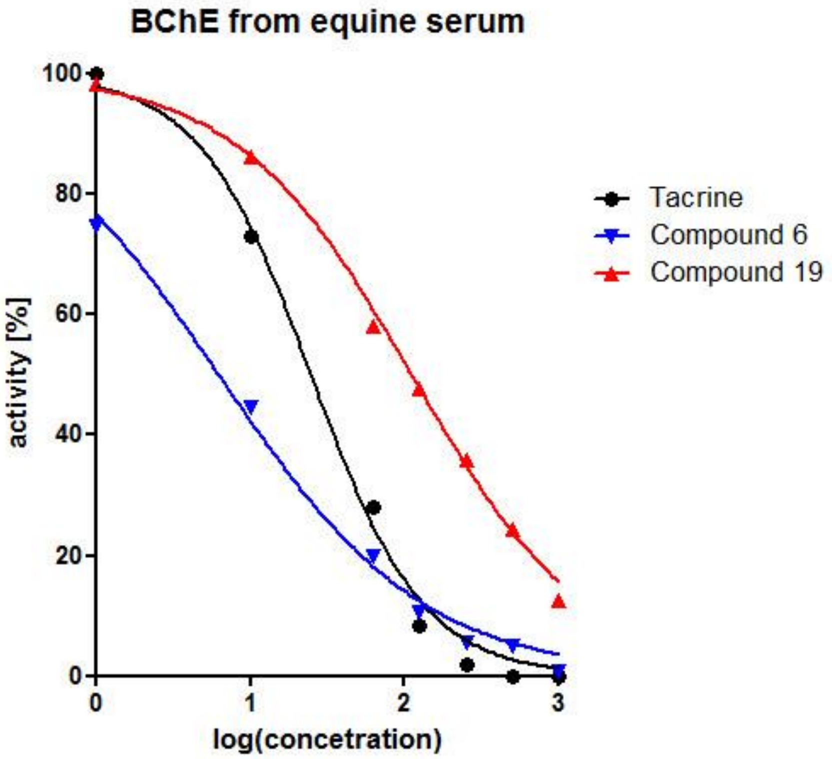

Figure 4. Inhibition curves of compounds **6** and **19** against BChE.

^{1}H NMR Compound **6**

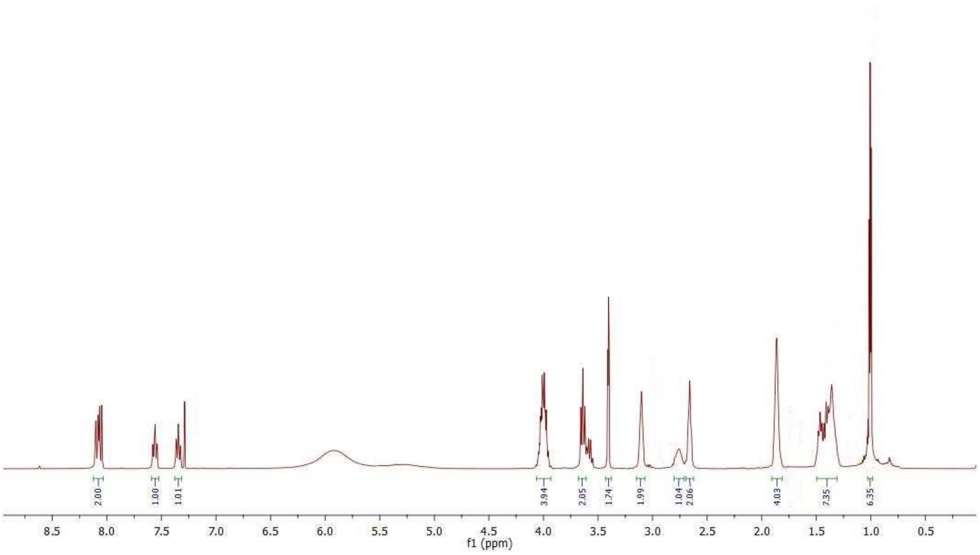

^{13}C NMR Compound **6**

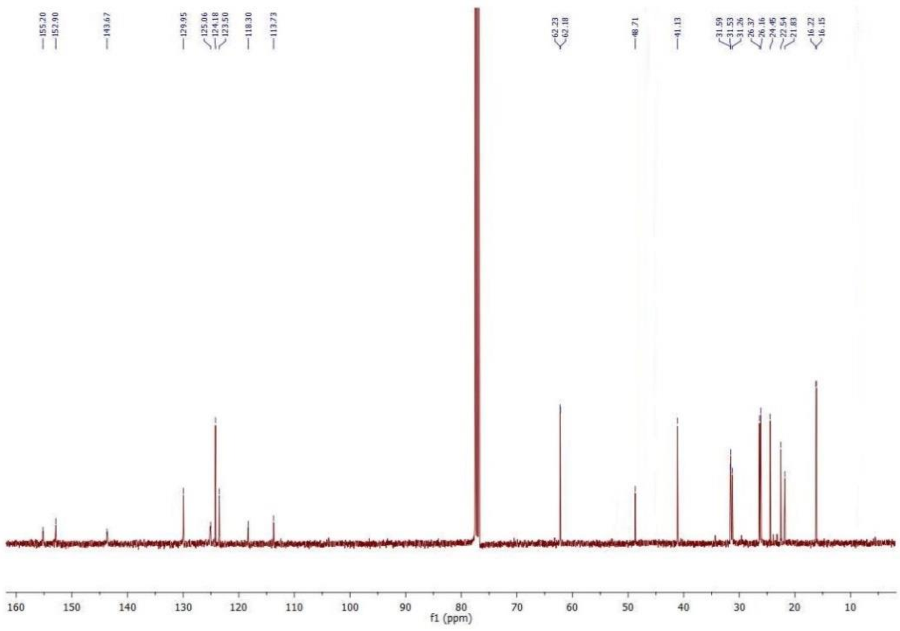

MS Compound **6**

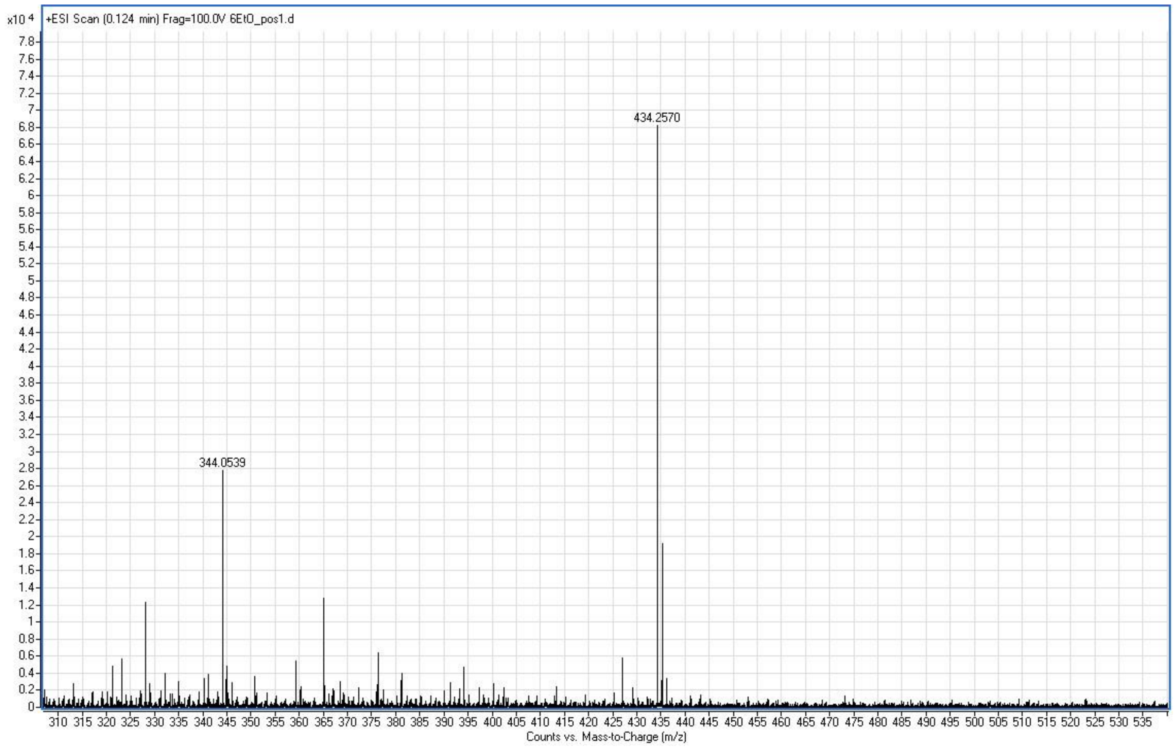

^{31}P NMR Compound **6**

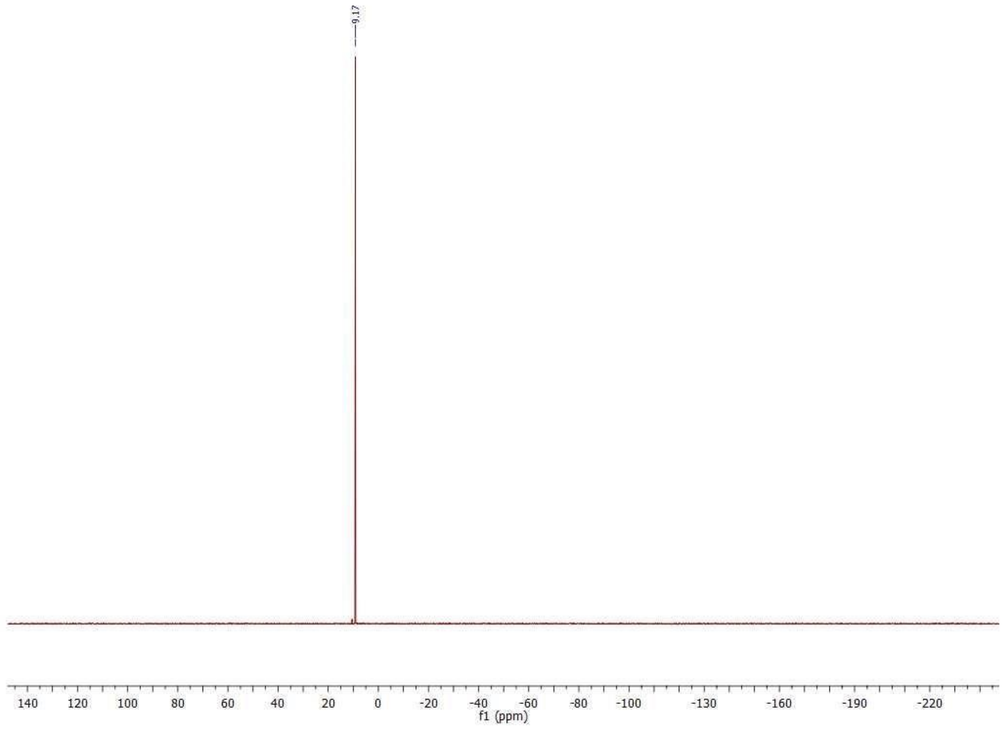

^{1}H NMR Compound **21**

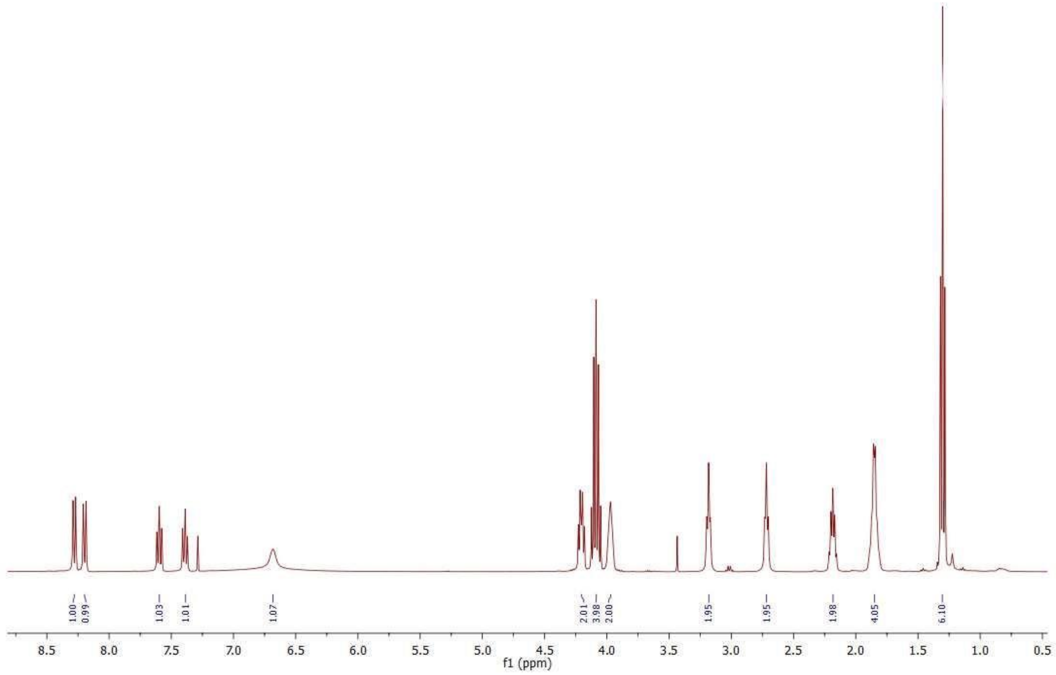

^{13}C NMR Compound **21**

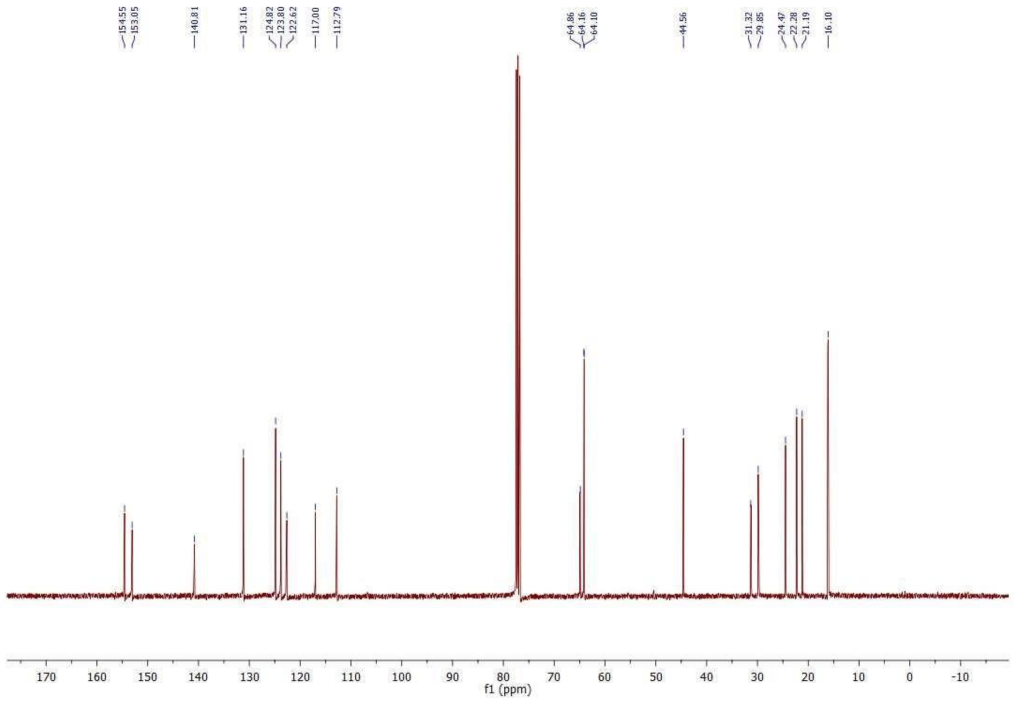

MS Compound **21**

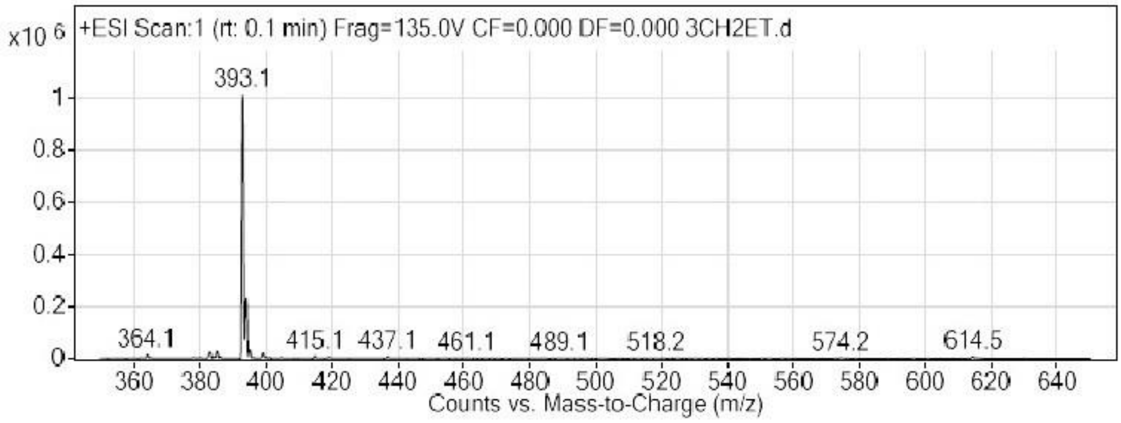

^{31}P NMR Compound **21**

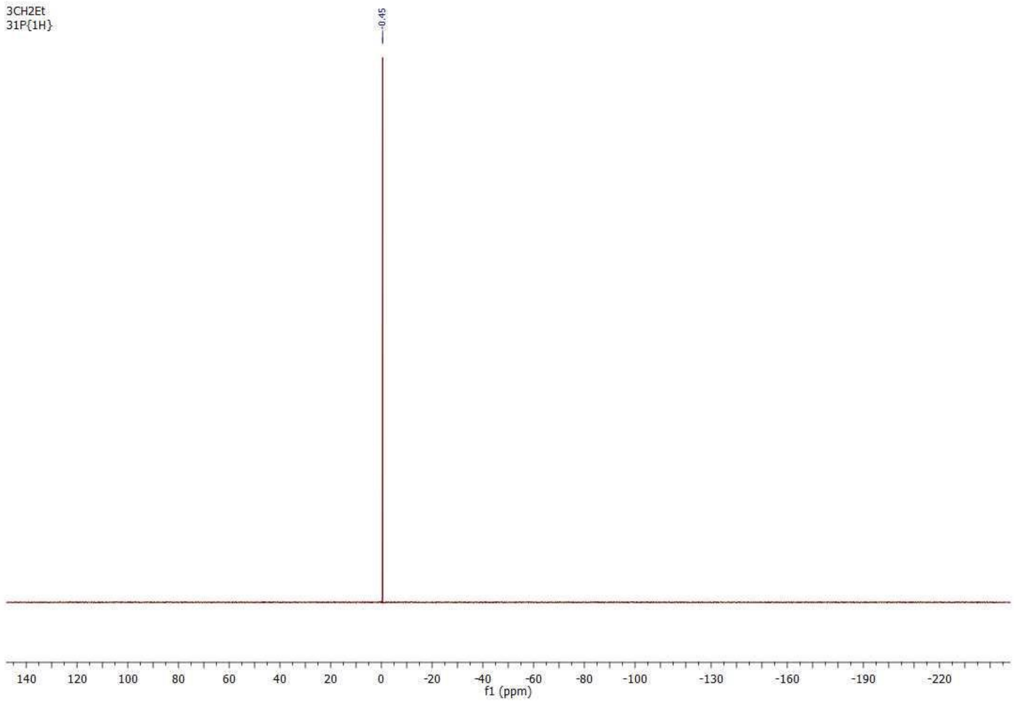

Supplement: Supplemental Material [file IENZ_A_2045591_SM4353.pdf]
